# Effect of deep learning reconstruction on the assessment of pancreatic cystic lesions using computed tomography

**DOI:** 10.1007/s12194-024-00834-6

**Published:** 2024-08-15

**Authors:** Jun Kanzawa, Koichiro Yasaka, Yuji Ohizumi, Yuichi Morita, Mariko Kurokawa, Osamu Abe

**Affiliations:** Department of Radiology, University of Tokyo Hospital, Bunkyo-ku, Tokyo Japan

**Keywords:** Pancreatic cyst, Deep learning reconstruction, Filtered back projection, Computed tomography

## Abstract

This study aimed to compare the image quality and detection performance of pancreatic cystic lesions between computed tomography (CT) images reconstructed by deep learning reconstruction (DLR) and filtered back projection (FBP). This retrospective study included 54 patients (mean age: 67.7 ± 13.1) who underwent contrast-enhanced CT from May 2023 to August 2023. Among eligible patients, 30 and 24 were positive and negative for pancreatic cystic lesions, respectively. DLR and FBP were used to reconstruct portal venous phase images. Objective image quality analyses calculated quantitative image noise, signal-to-noise ratio (SNR), and contrast-to-noise ratio (CNR) using regions of interest on the abdominal aorta, pancreatic lesion, and pancreatic parenchyma. Three blinded radiologists performed subjective image quality assessment and lesion detection tests. Lesion depiction, normal structure illustration, subjective image noise, and overall image quality were utilized as subjective image quality indicators. DLR significantly reduced quantitative image noise compared with FBP (*p* < 0.001). SNR and CNR were significantly improved in DLR compared with FBP (*p* < 0.001). Three radiologists rated significantly higher scores for DLR in all subjective image quality indicators (*p* ≤ 0.029). Performance of DLR and FBP were comparable in lesion detection, with no statistically significant differences in the area under the receiver operating characteristic curve, sensitivity, specificity and accuracy. DLR reduced image noise and improved image quality with a clearer depiction of pancreatic structures. These improvements may have a positive effect on evaluating pancreatic cystic lesions, which can contribute to appropriate management of these lesions.

## Introduction

Pancreatic cystic lesions consist of various entities ranging from benign to precancerous [1]. They demonstrate a high incidence of 24.3% in autopsy series [2]. Advancements in imaging techniques make it possible to detect these lesions in asymptomatic patients [3]. The prevalence of incidental pancreatic cysts on imaging was reported to be 2.6% on 16-slice multidetector computed tomography (CT) and as high as 44.7% on magnetic resonance cholangiopancreatography [3, 4]. Intraductal papillary mucinous neoplasm (IPMN) is one of the most frequent pancreatic cystic neoplasms, accounting for approximately 25% [5]. IPMN is a precursor of pancreatic cancer [5]. Therefore, incidental detection of pancreatic cystic lesions is clinically important. Regarding the detectability of pancreatic lesions, magnetic resonance imaging (MRI) is superior to CT in contrast between cystic lesions and background parenchyma and is more sensitive, as described above. Conversely, CT is the primary modality of choice in assessing many diseases that originate in the abdomen, including pancreatic cystic lesions, because of its wide availability and fast acquisition time [1]. High spatial resolution and temporal resolution of CT enable depiction of detailed pancreatic anatomy and cyst morphology. Therefore, enhancing the detection sensitivity of pancreatic cystic lesions would demonstrate considerable practical merit.

Recently, deep learning applications have gained wide attention in the field of radiology [6]. Recent studies have revealed that deep learning allows not only imaging diagnosis [7] but also image processing [6]. Deep learning reconstruction (DLR) is one of such algorithms. DLR reduces noise and improves image quality without reducing spatial resolution [6]. Clinical application of DLR has also been investigated [8–11]. Studies on the liver have been frequently reported regarding abdominal imaging [8–10]. For example, DLR enhanced the image quality and conspicuity of hypovascular liver metastasis in contrast-enhanced CT [9]. Improved image quality by DLR contributed to improve hepatocellular carcinoma detection and interobserver agreement for liver imaging reporting and data system categories in abdominal dynamic contrast-enhanced CT [10]. Moreover, DLR reduced streak artifacts in abdominal CT performed without arm elevation and generated better-quality images [11]. We hypothesized that DLR can improve the detection performance or depiction of pancreatic cystic lesions because of its benefit in abdominal imaging. However, to the best of our knowledge, whether or not noise reduction achieved by DLR can facilitate pancreatic cystic lesion evaluation with CT remains unclear.

This study aimed to compare the image quality and detection performance of pancreatic cystic lesions between CT images reconstructed with DLR and filtered back projection (FBP).

## Materials and methods

Our Institutional Review Board approved this retrospective study, and the requirement for obtaining written informed consent was waived.

### Patients

We searched the picture archiving and communication system (PACS) for all consecutive patients with and without pancreatic cystic lesions who underwent contrast-enhanced CT with portal venous phase from May 2023 to July 2023 and from June 2023 to August 2023, respectively. This study excluded two patients with pancreatic solid tumors (one pancreatic cancer and one neuroendocrine tumor) and two patients with pancreatic pseudocysts associated with pancreatitis because our study focused specifically on primary pancreatic cystic lesions. Our study included 54 patients (34 males and 20 females; mean age: 67.7 ± 13.1 years). Among eligible subjects, 30 were diagnosed as positive for pancreatic cystic lesions and 24 as negative. The presence of pancreatic cystic lesions was confirmed by CT in 12 patients and both CT and T2-weighted sequences of MRI in 18 patients. The absence of pancreatic cystic lesions was confirmed by T2-weighted sequences of MRI.

### CT imaging

All patients underwent CT examination with Aquilion ONE (Canon Medical Systems, Otawara, Japan). CT scanning parameters were as follows: tube voltage of 120 kVp; tube current of automatic tube current modulation was used with standard deviation (SD) set at 13.0, the helical pitch of 0.8125:1, and gantry rotation time of 0.5 s. Iodinated contrast media at 600 mgI/kg was intravenously injected using a power injector, and the portal venous phase was obtained 70–90 s after starting the injection. Raw CT data were processed using two different reconstruction algorithms: FBP with the reconstruction kernel of FC03 and DLR (advanced intelligent clear-IQ Engine with body sharp standard). The following image reconstruction parameters were same for FBP and DLR: field of view of 350 mm (adjusted to body size) and slice thickness/interval of 3/3 mm.

CT images were anonymized and exported from the PACS in Digital Imaging and Communications in Medicine format.

### Quantitative image analysis

Quantitative image analyses were conducted using ImageJ software (RRID: SCR_003070). A radiologist (radiologist A with 2 years of post-residency experience in imaging diagnosis) placed regions of interest (ROIs) of approximately 10-mm diameter on the pancreatic cystic lesion, head of the pancreas (right side of the left border of the superior mesenteric vein), and aorta at the level of the origin of the superior mesenteric artery under the supervision of a senior radiologist (radiologist B with 13 years of post-residency experience in imaging diagnosis). If there were multiple lesions, the largest lesion was selected. The largest ROI possible was placed if lesions were smaller than 10 mm in diameter. The peripheral part of the lesion was avoided when placing ROIs to reduce the partial volume effect. ROIs were placed on DLR images, and the same ROIs were copied to the FBP image to ensure that the location, size, and shape of the ROIs were the same between DLR and FBP. The mean and SD of the CT attenuation were recorded. SD of the aorta was used as an image noise indicator. The signal-to-noise ratio (SNR) and contrast-to-noise ratio (CNR) were calculated as follows: *SNR* = *CTpancreas*/*noise* (1).

*CNR* = (*CTpancreas−CTlesion*)/*noise* (2),

where *CTpancreas* denotes the mean CT attenuation of the pancreas and *CTlesion* indicates mean CT attenuation of cystic lesions.

### Lesion detection and qualitative image analysis

Three other radiologists (readers 1, 2, and 3 with 7, 3, and 1 year of post-residency experience in imaging diagnosis, respectively) were involved. They were blinded to the patient background information and the image reconstruction algorithm. A single image set was evaluated at a time (i.e., not in a side-by-side way) with ImageJ software. The three radiologists independently evaluated the image sets.

Readers assessed the presence of pancreatic cystic lesions for each of the three pancreatic segments in the lesion detection test (head of pancreas: right side of the left border of superior mesenteric vein, body: between the left border of superior mesenteric vein and left border of aorta, and tail: left side of the left border of aorta) and recorded locations and confidence scores (4 = definitely present, 3 = probably present, 2 = uncertain for the presence or absence, and 1 = no lesion). If there were multiple lesions, they assessed the largest lesions in each segment. Radiologist B randomized image sets to reduce recall bias by avoiding the overlap of different reconstruction algorithms of a single patient within a single part, and a 2-week interval was set between each part.

The same three radiologists evaluated the images after the lesion detection test, in terms of the following: (1) lesion depiction (largest lesions in each segment were assessed) (4 = clear depiction, 3 = slightly blurred, 2 = moderately blurred, and 1 = unrecognizable); (2) structure depiction (pancreatic parenchyma and main pancreatic duct) (4 = clear depiction, 3 = slightly blurred, 2 = moderately blurred, and 1 = unrecognizable); (3) subjective image noise on a 4-point scale (4 = less noise, 3 = standard noise, 2 = more than standard noise, and 1 = severe noise); (4) overall image quality on a 5-point scale (5 = excellent, 4 = better than standard, 3 = standard, 2 = worse than standard, and 1 = poor). Radiologist B randomized all image sets for qualitative image analyses. These evaluation methods were adapted from previous reports evaluating DLR on abdominal imaging [10, 11].

### Statistical analysis

Statistical analyses were performed with EZR version 1.37 (https://www.jichi.ac.jp/saitama-sct/SaitamaHP.files/statmed.html) [12], which is a graphical user interface of R version 2.4–0 (R Foundation for Statistical Computing, Vienna, Austria RRID: SCR_001905).

Paired *t* tests were used to compare results for quantitative analyses between DLR and FBP. Diagnostic performance for lesion detection was calculated using the area under the receiver operating characteristic curve (AUC) and was compared between DLR and FBP using the DeLong test. Sensitivity, specificity, and accuracy were compared between DLR and FBP using McNemar’s test. Sensitivity, specificity, and accuracy were calculated using a confidence score cut-off of 4 (definitely present) to evaluate the confident detection of lesions. This means that lesions scored as 4/3 or less were considered positive/negative detections for these calculations. Qualitative analysis results were compared between DLR and FBP using the Wilcoxon signed rank test. A *p* value of 0.050 indicated a statistically significant difference for all tests.

## Results

### Quantitative image analysis

Table 1 shows the quantitative image analyses. Quantitative image noise was significantly reduced in DLR compared with FBP (*p* < 0.001). SNR and CNR were significantly higher in DLR compared with FBP (*p* < 0.001).

**Table 1:** Results of quantitative image analysis

	Result (mean ± SD)		Comparison (*p*)
	FBP	DLR	FBP *vs.* DLR
Noise	19.23 ± 2.23	8.28 ± 0.92	< 0.001*
SNR	4.75 ± 1.17	10.95 ± 2.45	< 0.001*
CNR	3.65 ± 1.24	8.48 ± 2.63	< 0.001*

*SD* standard deviation, *SNR* signal-to-noise ratio, *CNR* contrast-to-noise ratio, *FBP* filtered back projection, *DLR* deep learning reconstruction

*Statistically significant difference (*p* < 0.050). Comparisons were performed with the paired *t* test

### Lesion detection

Table 2 shows the lesion detection results. There were no statistically significant differences in AUC, sensitivity, specificity, and accuracy between DLR and FBP. For readers 1 and 2, actual values of these metrics in DLR were slightly higher than or equal to FBP. For reader 3, DLR showed slightly higher or equal values in sensitivity and accuracy, but slightly lower values in AUC and specificity compared to FBP.

**Table 2:** Results of lesion detection

Reader	FBP	DLR	Comparison (*p*)
AUC (95% confidence intervals)			
1	0.885 (0.824–0.946)	0.900 (0.841–0.959)	0.510
2	0.835 (0.763–0.907)	0.888 (0.828–0.949)	0.140
3	0.855 (0.782–0.928)	0.851 (0.776–0.927)	0.900
Sensitivity (95% confidence intervals)			
1	0.717 (0.565–0.840)	0.761 (0.612–0.874)	0.724
2	0.761 (0.612–0.874)	0.761 (0.612–0.874)	1.000
3	0.696 (0.542–0.823)	0.717 (0.565–0.840)	1.000
Specificity (95% confidence intervals)			
1	0.957 (0.902–0.986)	0.974 (0.926–0.995)	0.617
2	0.888 (0.816–0.939)	0.931 (0.869–0.970)	0.182
3	0.948 (0.891–0.981)	0.940 (0.880–0.975)	1.000
Accuracy (95% confidence intervals)			
1	0.889 (0.830–0.933)	0.914 (0.859–0.952)	1.000
2	0.852 (0.788–0.903)	0.883 (0.823–0.928)	0.359
3	0.877 (0.816–0.923)	0.877 (0.816–0.923)	0.773

Comparisons were performed with the DeLong test for AUC and McNemar’s test for sensitivity, specificity, and accuracy

*AUC* area under the receiver operating characteristic curve, *FBP* filtered back projection, *DLR* deep learning reconstruction

**Fig. 1:**
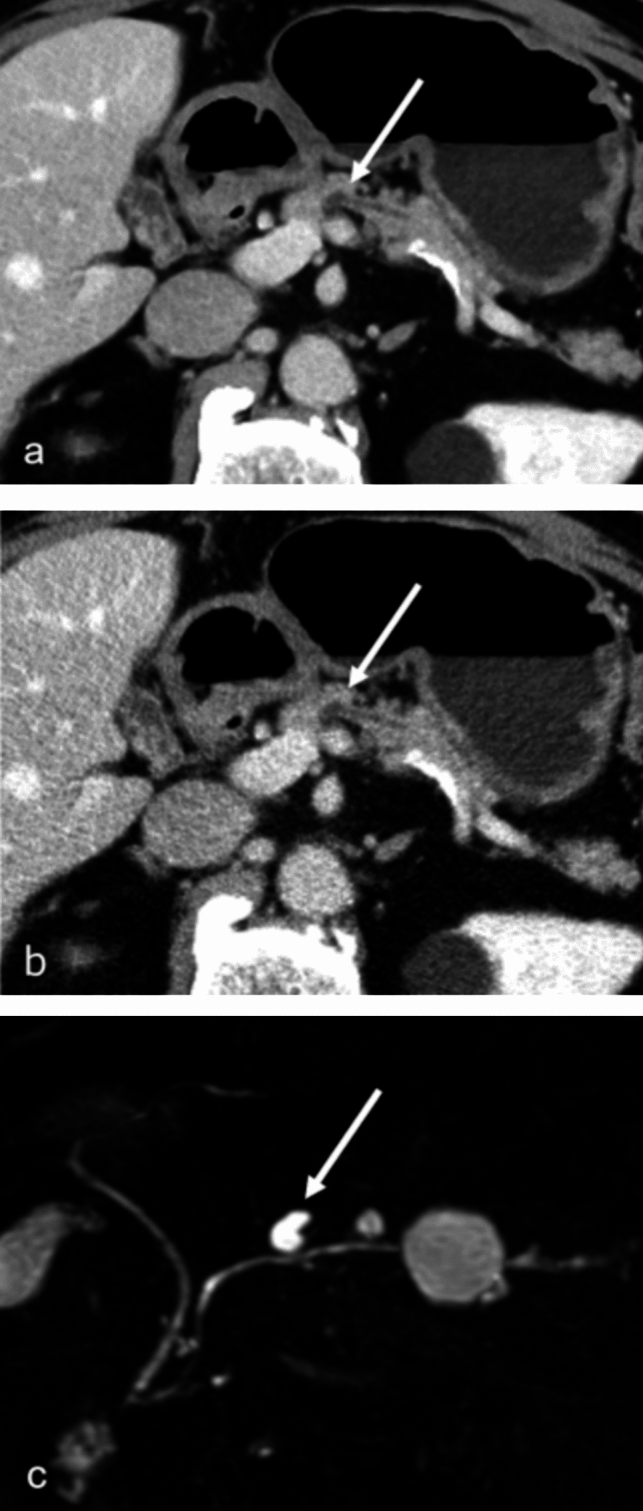
CT images of a male in his 70 s. Images were reconstructed with deep learning reconstruction (DLR) (**a**) and filtered back projection (FBP) (**b**) in the portal venous phase. Magnetic resonance cholangiopancreatography (MRCP) was taken approximately 6 months before CT (**c**)

**Fig. 2:**
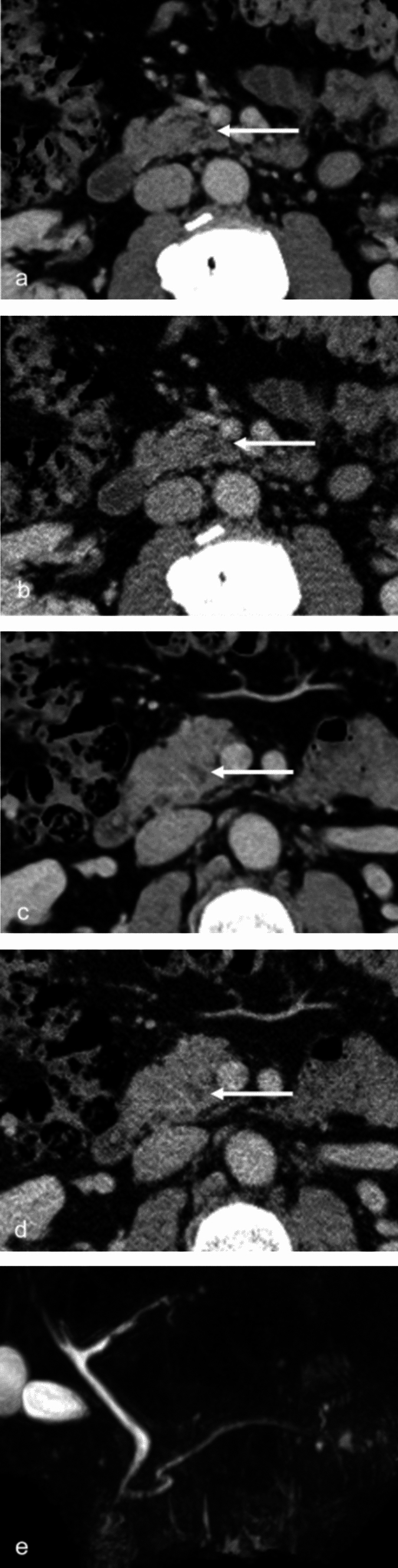
CT images of a male in his 50 s. Images were reconstructed with deep learning reconstruction (DLR) (**a, c**) and filtered back projection (FBP) (**b, d**) in the portal venous phase. Magnetic resonance cholangiopancreatography (MRCP) was taken approximately 3 weeks after CT (**e**)

Figures 1 and 2 show representative cases. Figure 1 demonstrates a pancreatic cystic lesion in a male patient in his 70s. The lesion, abutting the main pancreatic duct, was more clearly depicted on DLR (Fig. 1a) compared to FBP (Fig. 1b). Three readers scored lesion depiction as 4, 4, and 4 on DLR, and 4, 3, and 3 on FBP. Other image quality indicators (pancreatic parenchyma depiction/main pancreatic duct depiction/subjective image noise/overall image quality) were scored as 4/3/4/5, 4/3/4/4, and 4/3/4/4 on DLR, and 2/3/2/3, 3/2/3/3, and 3/2/2/3 on FBP by the three readers. The lesion’s presence was confirmed on MRCP (Fig. 1c).

Figure 2 shows images from a male patient in his 50s. A low-density area in the pancreatic head, suspected to be fatty infiltration, was misinterpreted as a pancreatic cystic lesion by reader 2 on FBP (Fig. 2b) with a confidence score of 4, but correctly ruled out on DLR (Fig. 2a) with a confidence score of 1. Reader 3 misinterpreted another low-density area on FBP (Fig. 2d) with a confidence score of 3, but ruled it out on DLR (Fig. 2c) with a confidence score of 1. Reader 1 correctly ruled out both on DLR and FBP. Other image quality indicators were scored as 4/3/4/4, 4/3/4/5, and 4/2/4/3 on DLR, and 2/2/2/3, 3/2/2/3, and 4/2/2/4 on FBP by the three readers. The absence of pancreatic cystic lesions was confirmed on MRCP (Fig. 2e).

### Qualitative image quality analyses

Table 3 shows the subjective image quality analyses. DLR scored significantly higher than FBP in all image quality indicators (lesion depiction, pancreatic parenchyma depiction, main pancreatic duct depiction, image noise, and overall image quality (*p* ≤ 0.029).

**Table 3:** Results of subjective image analyses

	Reader	FBP	DLR	Comparison (*p*)
Depiction of structures (score 4/3/2/1)				
Lesion	1	4/14/19/6	24/17/2/0	< 0.001*
	2	0/18/24/1	29/14/0/0	< 0.001*
	3	13/19/10/1	18/20/5/0	0.029*
Parenchyma	1	2/20/30/2	43/8/3/0	< 0.001*
	2	0/40/14/0	33/21/0/0	< 0.001*
	3	32/19/3/0	43/11/0/0	0.003*
MPD	1	1/7/38/8	11/16/26/1	< 0.001*
	2	0/10/39/5	16/24/13/1	< 0.001*
	3	1/20/28/5	5/27/20/2	< 0.001*
Image noise (score 4/3/2/1)				
	1	0/9/40/5	45/7/2/0	< 0.001*
	2	0/12/41/1	48/5/1/0	< 0.001*
	3	0/15/38/1	46/8/0/0	< 0.001*
Overall image quality (score 5/4/3/2/1)				
	1	0/5/23/24/2	38/13/2/1/0	< 0.001*
	2	0/0/40/14/0	26/23/5/0/0	< 0.001*
	3	0/9/34/11/0	2/28/22/2/0	< 0.001*

Comparisons were performed between DLR and FBP using Wilcoxon signed-ranks tests

Numbers of patients for each score are shown. *FBP* = filtered back projection, *DLR* deep learning reconstruction. lesion pancreatic cystic lesions. parenchyma: pancreatic parenchyma. *MPD* main pancreatic duct

*A statistically significant difference (*p* < 0.05)

## Discussion

Our study revealed that DLR reduced objective image noise and improved SNR and CNR compared with FBP. Further, subjective image scores of structure depictions (cystic lesions, pancreatic parenchyma, and main pancreatic duct), noise, and overall image quality were significantly higher in DLR than in FBP. On the other hand, there was no statistically significant difference in pancreatic cystic lesion detection between DLR and FBP, though DLR tended to show slightly higher values in performance metrics in two out of three readers’ evaluations.

The reduction of objective image noise by DLR has been reported both in phantom analysis and clinical studies [8]. Our study revealed that DLR reduced the mean image noise by 56.9% compared with FBP. This result is almost equivalent to previous reports [8], which confirms positive effect of DLR on noise reduction.

Evaluating detailed structures is important in assessing pancreatic cystic lesions to determine the necessities of surgical management [1]. Our study revealed that DLR images improved depiction of lesions and normal pancreatic structures. Further, DLR demonstrated a reduction in objective image noise. These results suggest that DLR facilitates clinical decision-making by increasing visibility of detailed structures. The strength of our study is the use of portal venous phase images in image assessment. Portal venous phase images are routinely used for abdominal surveys [13]. Thus, our results suggest that DLR enables the improved visualization of pancreatic cystic lesions in daily clinical practice.

Regarding the detection of pancreatic cystic lesions, DLR and FBP demonstrated comparable performance, with slight differences observed in performance metrics. A previous study demonstrated no significant improvement in lesion detection with DLR, which aligns with our study [14]. On the other hand, another study demonstrated significant improvement [10]. This discrepancy implies that the impact of DLR on lesion detection may vary depending on the type of lesions and organs involved. Given the lack of comprehensive analysis on the performance of DLR on various lesions, future research is needed to investigate the factors influencing its effectiveness in lesion detection. Still, the difference in accuracy between DLR and FBP ranged from 0% to 3.1% across three readers. It can be inferred that DLR could potentially enhance the detection rate of pancreatic cysts on CT by up to about 3% compared to FBP.

A previous study revealed a 62% sensitivity of CT for detecting pancreatic cysts [15]. Our sensitivity results were higher than these data for both FBP and DLR, which can be attributed to the method of lesion detection used in our study. Our study divided the pancreas into three segments, and the presence of cystic lesions was assessed in each segment. This may have resulted in a detailed assessment and improved sensitivity.

This study had some limitations. First, this retrospective study was conducted at a single institution with a relatively small population. Second, our study focused on pancreatic cystic lesions; thus, other pancreatic lesions were not included. Finally, this study was conducted using a deep-learning reconstruction algorithm produced by a vendor. Our results would not necessarily apply to similar algorithms available from other vendors. These factors may limit the generalizability of our results. Future studies are required to investigate the utility of DLR with large sample sizes, various pancreatic pathologies, and different algorithms.

In conclusion, DLR reduced image noise and improved image quality with a clearer pancreatic structure depiction. Our results suggest that DLR has a positive effect on evaluating pancreatic cystic lesions. These improvements can contribute to appropriate management of pancreatic cystic lesions with CT, which is widely available compared to MRI.

## Data Availability

The datasets of this study are available from the corresponding author upon reasonable request.
